# A Case of Transanal Total Mesorectal Excision Using the Senhance Digital Laparoscopy System for Rectal Cancer

**DOI:** 10.70352/scrj.cr.24-0024

**Published:** 2025-02-01

**Authors:** Yasuhiro Ishiyama, Yasumitsu Hirano, Yume Minagawa, Misuzu Yamato, Sohei Akuta, Akihito Nakanishi, Takatsugu Fujii, Naoto Okazaki, Chikashi Hiranuma, Isamu Koyama

**Affiliations:** Department of Gastroenterological Surgery, Saitama Medical University International Medical Center, Hidaka, Saitama, Japan

**Keywords:** senhace surgical system, transanal total mesorectal excision, rectal cancer

## Abstract

**INTRODUCTION:**

This case report presents the world’s first transanal total mesorectal excision (taTME) using the Senhance Digital Laparoscopy System for rectal cancer. Senhance has been gaining attention as an advanced surgical robot, providing better visualization, stable dissection, and reduced surgeon fatigue compared to conventional methods.

**CASE PRESENTATION:**

A 68-year-old woman underwent Senhance-assisted taTME for a rectal tumor located 4 cm from the anal verge. The procedure was completed safely with a total operation time of 209 minutes, a cockpit time of 85 minutes, and a blood loss of 40 mL. Pathological evaluation confirmed complete resection (R0) with no residual cancer or lymph node metastases. The patient was discharged on postoperative day 7 without complications.

**CONCLUSIONS:**

This case demonstrates the potential advantages of Senhance-assisted taTME, including improved visualization, stable dissection, and reduced surgeon fatigue. Further studies are needed to evaluate long-term outcomes and establish the role of this technique in rectal cancer surgery.

## Abbreviations

ESD
: endoscopic submucosal dissection
taTME
: transanal total mesorectal excision

## INTRODUCTION

The incidence of rectal cancer has increased from 27% in 1995 to 31% in 2019 and is expected to continue rising.^1)^ Meanwhile, transanal total mesorectal excision (taTME) for low rectal cancer, first reported by Lacy et al.^2)^ has been shown to provide excellent visualization for tumors located in the pelvic floor,^2)^ improving surgical outcomes.^3)^ However, technical skill requirements and instrument limitations still exist.

**Fig. 1:**
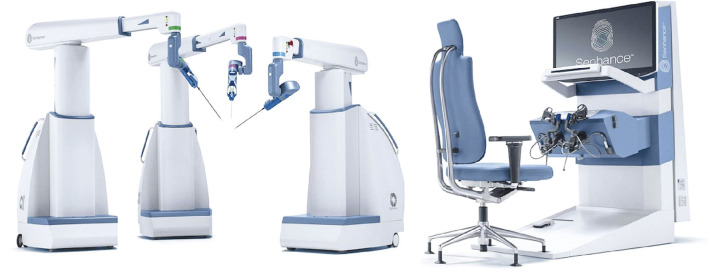
Senhance Digital Laparoscopy System.

The Senhance Digital Laparoscopy System (hereafter Senhance, Asensus Surgical Inc., Durham, NC, USA, **Fig. 1**) has garnered attention as a surgical robot following da Vinci. Senhance is a state-of-the-art surgical robot that offers higher quality and stability than laparoscopic surgery through features such as tremor filtration, motion scaling, haptic feedback, eye-tracking camera control, 3D vision, and a unique cockpit. Senhance uses the same devices as those used in laparoscopic surgery, including endoscopic-style instruments, facilitating an easy technical transition from laparoscopic surgery and allowing for instrument reusability.^4)^ This system has also been reported to be cost-effective compared to other automated systems due to its reusable instruments.^4)^ While there are several reports on colorectal cancer surgery using Senhance,^5)^ there are currently no reports on taTME using Senhance. We report here the world’s first Senhance-assisted taTME for rectal cancer surgery performed in June 2024.

## CASE PRESENTATION

A 68-year-old woman with a BMI of 23.5 kg/m^2^ presented to our hospital with abdominal pain. Colonoscopy revealed a 20 mm tumor at 4 cm from the anal verge. Endoscopic submucosal dissection (ESD) was performed by the gastroenterology department. Pathological examination diagnosed well-differentiated adenocarcinoma, Lv1 (D2-40), V0 (EVG), BD2, HM0, VM1. Due to positive margins, the patient was referred to surgery for additional resection. Contrast-enhanced CT of the chest and abdomen showed no significant lymph node enlargement or distant metastases, so surgery was planned.

### Surgical technique

#### Abdominal approach

Under general anesthesia, the patient was positioned with the head 15 degrees down and 15 degrees to the right. A 3 cm vertical incision was made at the umbilicus to install the EZ Access, and a 12 mm port was placed in the right lower abdomen. The surgery was performed using the Single Incision Laparoscopic Surgery plus one port technique, as we have previously reported.^6)^

#### Transanal approach

**Fig. 2:**
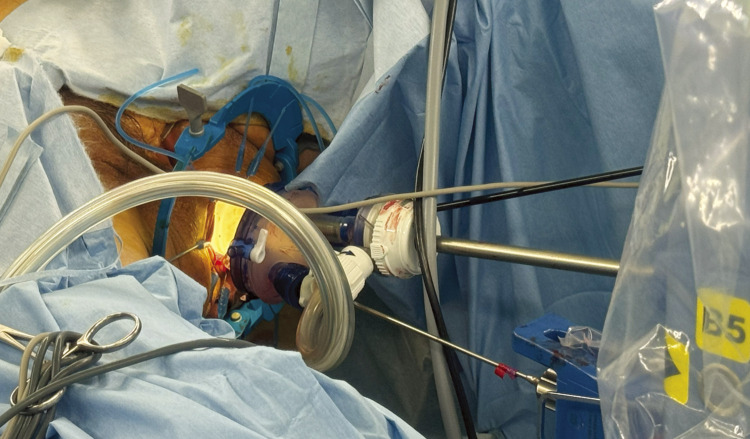
Transanal approach using the Senhance Digital Laparoscopy System using 12, 5, 5, 5 mm ports under GelPOINT.

**Fig. 3:**
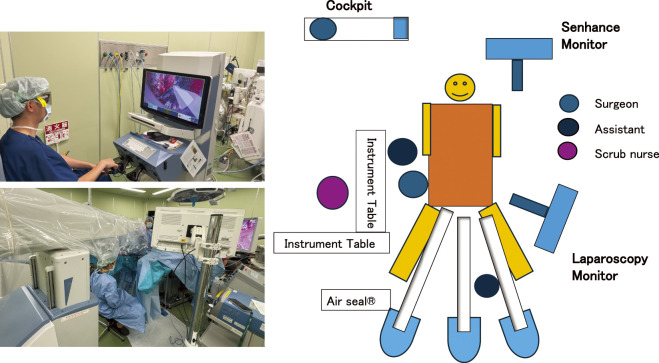
Setting of the Senhance Digital Laparoscopy System. All three robotic arms of the Senhance system were positioned between the patient’s legs. The surgeon operated the system remotely from the Cockpit console.

A self-fixing anal retractor (Lone Star CooperSurgical, Trumbull, CT, USA) was installed to expose the anal canal, and a GelPOINT (Applied Medical, Rancho Santa Margarita, CA, USA) was attached. Four ports were placed (**Fig. 2**). A two-team approach with abdominal and anal operations was initiated (**Fig. 3**).

The Senhance Digital Laparoscopy System was used with three robotic arms. A 30-degree videoscope (Storz), a 5 mm Monopolar L-shaped hook Instrument (Senhance), and a 3 mm Bipolar Instrument (Senhance) were attached to Senhance. The fourth port was placed as an assistant port. The Senhance arms were positioned from the patient’s anal side and docked. (**Fig. 3**) The assistant was positioned on the anal side (**Fig. 3**) while the Senhance operator performed the procedure from the cockpit.

**Fig. 4:**
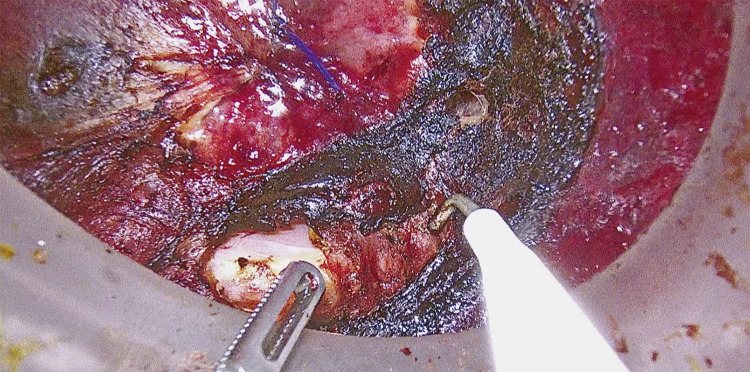
The intraperitoneal and posterior rectal walls rendezvoused while Senhance-assisted taTME surgical procedure.

A smoke evacuation system (AirSeal System Surgiquest, Milford, CT, USA) was used. The surgery began with CO_2_ insufflation at 15 mmHg. Marking was made 2 cm anal to the tumor, and a purse-string suture was performed to close the rectum. The rectal mucosa was incised through the full thickness of the rectum. The longitudinal muscle was exposed circumferentially. The longitudinal muscle was incised, exposing the prostate on the anterior wall (**Fig. 4**). On the posterior wall, the endopelvic fascia was used as a landmark for incision. A rendezvous was performed with the abdominal cavity. The rendezvous point for the anterior wall of the rectum is at the peritoneal reflection, while for the posterior wall, it is at the S3 level. The lateral walls are dissected after the anterior and posterior walls are rendezvoused and are approached from the intraperitoneal side. The specimen was extracted through the umbilical incision site. Anastomosis was performed using the single stapler technique.

The total operation time was 209 minutes, with a cockpit time of 85 minutes, and blood loss was 40 mL. Transanal and pelvic drains were placed. There were no intraoperative complications.

#### Postoperative course

The postoperative course was uneventful, and the patient was discharged on postoperative day 7. Pathological evaluation showed complete resection (R0) with no residual cancer and no lymph node metastases.

## DISCUSSION

Senhance has expanded to various surgeries, with reports of safety and efficacy in urological, abdominal, thoracic, and gynecological fields, and is reported as an alternative to da Vinci for robot-assisted surgery.^7)^ Our hospital has also reported stable surgical results for colorectal cancer.^5)^ However, there are currently no reports on single-incision surgery or taTME. Nevertheless, our hospital has performed surgical techniques applying RPS with Senhance, and by leveraging this experience, we have introduced the world’s first Senhance-assisted taTME for rectal cancer.

Recently, there have been reports of taTME using the da Vinci platform for rectal cancer surgery, particularly noting improved dexterity in bilateral dissection.^8)^ Compared to conventional laparoscopic surgery, the surgical field is more stable. On the other hand, the 8 mm ports have been reported to cause interference during extracorporeal manipulation. Furthermore, da Vinci still has cost issues compared to laparoscopic surgery.^9)^

Senhance-assisted taTME allows the surgeon to perform the procedure from a relaxed position in the cockpit (**Fig. 2**). In conventional taTME, the surgeon often sits on the anal side of the patient, looking up, so performing the procedure from a relaxed position is expected to reduce muscular strain on the surgeon. Moreover, the 3D system provides depth perception, making it suitable for procedures like taTME that involve operating in narrow spaces. Stable dissection could be performed without hand tremors.

In conventional taTME procedures, the assistant is often required to maintain an ergonomically challenging posture while holding the scope, which can lead to significant physical strain. In contrast, Senhance-assisted taTME alleviates this issue for the assistant. The primary responsibility of the assistant in this robotic approach is instrument exchange, allowing them to remain seated during the procedure. This ergonomic advantage suggests that Senhance-assisted taTME may be a less physically demanding technique for the surgical assistant.

Furthermore, the transition from Senhance-assisted taTME to normal laparoscopic taTME can be achieved simply by disengaging the Senhance arms, ensuring safety. The problem of port interference observed during da Vinci-assisted taTME was mitigated in the Senhance system through the use of 3 mm forceps, which are smaller than their 5 mm counterparts. This reduction in instrument size facilitated decreased interference between ports.

On the other hand, the Senhance system requires adjustments to the patient’s positioning height to avoid arm interference when placing three arms between the legs due to the large size of its arms. In addition, the patient’s legs were raised higher than usual, above the abdominal level, using a levitator.

Given that patient morphology can significantly influence arm positioning, comprehensive preoperative simulation by the surgical team is crucial for determining optimal arm and monitor placement. Furthermore, operating table height adjustments were required during both anterior and posterior rectal wall dissection.

Although these limitations exist in the current iteration of the Senhance system, its technology continues to evolve. Future developments are anticipated to address these technical constraints.

Additionally, there are reports that Senhance does not depend on laparoscopic surgery experience, and even surgeons with little experience can adapt quickly,^10)^ suggesting that the introduction of Senhance-assisted taTME could be considered as an option in the future.

## CONCLUSIONS

We report our experience with performing taTME for rectal cancer using Senhance. While the da Vinci Surgical System currently dominates the surgical robotics market, the introduction of alternative platforms such as the Senhance Digital Laparoscopy system may contribute to the overall advancement of robotic surgery.

## ACKNOWLEDGMENTS

We would like to thank Ms. Kelli Armstrong, Clinical Strategy Director at Asensus Surgical, Inc., for her valuable assistance in editing a draft of this manuscript.

## DECLARATIONS

### Funding

Not Applicable.

### Authors’ contributions

Y. I, and Y.H designed the study and performed the experiments; Y.I, Y.M, T.F, and N.O wrote the manuscript.

Y.H, C.H, M.Y, and S.A drafted the original manuscript.

Y.H and I.K supervised the conduct of this study.

All authors have read and approved the manuscript, and they are responsible for the manuscript.

### Availability of data and materials

Not applicable.

### Ethics approval and consent to participate

All study participants provided informed consent, and the study was approved by the Institutional Review Board of Saitama Medical University International Medical Center (IRB number 2023-1895).

### Consent for publication

Written informed consent for publication of this study, including the use of medical data and any accompanying images, was obtained from all participating patients.

### Disclosure statement

Yasumitsu Hirano: Consultant (self) (Asensus Surgical), Honoraria (self) (Asensus Surgical). Isamu Koyam: Consultant (Self): (Asensus Surgical), Honoraria (self) (Asensus Surgical).

Drs Y Ishiyama, Y Minagawa, M Yamato, S Akuta, A Nakanishi, T Fujii, N Okazaki, and C Hiranuma have no conflicts of interest or financial ties to disclose.
